# Novel and Predominant Pathogen Responsible for the Enterovirus-Associated Encephalitis in Eastern China

**DOI:** 10.1371/journal.pone.0085023

**Published:** 2013-12-30

**Authors:** Lei Zhang, Jie Yan, David M. Ojcius, Huakun Lv, Ziping Miao, Yin Chen, Yanjun Zhang, Jvying Yan

**Affiliations:** 1 Zhejiang Provincial Center for Disease Control and Prevention, Hangzhou, Zhejiang, P.R.China; 2 Division of Basic Medical Microbiology, State Key Laboratory for Diagnosis and Treatment of Infectious Diseases, the First Affiliated Hospital, Zhejiang University School of Medicine, Hangzhou, Zhejiang, P.R. China; 3 Health Sciences Research Institute and School of Natural Sciences, University of California, Merced, Merced, California, United States of America; Washington University, United States of America

## Abstract

Enteroviruses (EV) have been increasingly identified as the causative agent for unknown etiological encephalitis in many parts of the world, but the long period surveillance for enterovirus-associated encephalitis (EAE) was not reported in China. From 2002-2012 in Zhejiang, Coxsackieviruses A9, B1, B2, B3, B4, B5; and echoviruses 3, 4, 6, 9, 14, 25, 30 were detected from the unknown etiological encephalitis cases, with coxsackievirus B4 been identified here for the first time. From 2002-2004 and 2010-2012, echovirus 30 was found to be the periodically predominant serotype for in the EAE. The molecular typing results showed that all the EV isolates from this study belonged to the human EV B (HEV B) family and were distributed in three clusters.

## Introduction

The enteroviruses (EVs) are RNA viruses comprise a large genus belonging to the *Picornaviridae*. According to the previous study, there are more than 100 serotypes that are divided into human enterovirus (EV) A to D can cause infections in humans [1]. Though most EV infections are mild or asymptomatic, it may also result in serious or even fatal disease, such as acute myocarditis and encephalitis [2]. Ashley et al determined that the EV was an important cause of encephalitis cases requiring hospitalization in California, and more EV serotypes have been identified in encephalitis cases from different parts of the world [3].

With high morbidity and mortality, encephalitis cause a large number of people hospitalization in China. Viruses are regarded as the most important etiological agents of encephalitis worldwide, while specific pathogen for the majority of the encephalitis cases has not been identified, depending on the existing diagnostic criteria [4]. Though Japanese encephalitis virus (JEV) was thought to be the most common cause of encephalitis, with the application of vaccine, the infection of JEV was well controlled, and only a small number of sporadic cases happened during recent years. Detection of EVs for the unknown etiological encephalitis may give clue for the etiology identification. On the other hand, there was no vaccine applied for most of the EVs, EV associated encephalitis outbreaks frequently in China, which calls for great attention, while the surveillance for the enterovirus associated encephalitis (EAE) was still almost neglected here [5,6].

In this study, patients with unknown etiological encephalitis in Zhejiang province were tested for the infections of the EVs by virus isolation and qRT-PCR. As continuous epidemiological surveillance is essential for identification of predominate EV serotypes or variants responsible for an outbreak and its disease pattern, this investigation was kept on conducting from 2002 to 2012. This study would provide the opportunity to describe the epidemiologic features of the EVE here, and their clinical manifestations were also discussed.

## Materials and Methods

### Surveillance designation and case definition

Four hospitals which located in the northern, southern, eastern and western part of Zhejiang province respectively were assigned to be the surveillance sites for the unknown etiological encephalitis. A case of encephalitis was defined as encephalopathy (depressed or altered level of consciousness lasting ≥24 hours, lethargy, or change in personality) requiring hospitalization and with ≥1 of the following findings: fever, seizure, focal neurologic findings, cerebrospinal fluid (CSF) pleocytosis, and electroencephalographic (EEG) or neuroimaging findings consistent with encephalitis. After excluding the infection of Japanese encephalitis virus and dengue virus, children (age less than 15 years) with unknown etiological encephalitis admitted to these hospitals were detected for enterovirus infection. Over the study period between 2002 and 2012, totally 1180 clinical specimens were collected from 1165 patients, including 826 CSF samples and 354 stool samples. The samples were stored at -70°C until used for analysis. This study was approved by the ethics committee of Zhejiang provincial Center for Disease Control and Prevention (CDC) and all participants provided written informed consent.

### Virus isolation and serotyping

Virus isolation is still regarded as the “gold standard” for EV identification. Isolation of an EV from associated body fluids and stool sample provides the strongest evidence of an enteroviral etiology. RD (Human rhabdomyosarcoma cells) and Hep-2 (Human epidermoid cancer cells) cell lines, which were maintained in MEM medium (Gibco, USA) supplemented with 2% fetal calf serum (FCS) (Gibco), 100 U/ml penicillin (Sigma, USA) and 100 mg/ml streptomycin (Sigma), in an atmosphere containing 5% CO_2_ at 37°C, were used for EV isolation in this study [2,7]. Following the isolation of the EV, the serotypic identity was determined by neutralization of infectivity with serotype specific anti-sera.

### Viral RNA extraction and RT-PCR identification

Viral RNA was extracted from these CSF and stool specimens by using the RNeasy Mini kit (Qiagen, USA) according to the manufacturer’s instructions. Briefly, 200µl of the specimens were mixed with 600 µl of RLT buffer and 6µl of β- mercaptoethanol and incubated for at least 1 min at room temperature. After the addition of 600µl of cold 70% ethanol, the mixture was vortexed and applied to a spin-column. After the washing steps, RNA was eluted in 30µl of RNase-free water. 

Real-time qRT-PCR reactions were performed by using the One-Step Realtime qRT-PCR Kit (TaKaRa, Japan). Each reaction mixture consisted of 10 pmol of the primers F: 5’-CTGYRGCGGAACCGACTAC-3’, R: 5’-ATTGTCACCATAAGCAGCCA-3’, 5 pmol of the probe P: 5’-FAM-TTGGGTGTCCGTGTTT-MGB-3’, and 4 μl of template RNA in a final volume of 50 μl. Real-time qRT-PCR reactions were performed according to the instructions of the manufacturer.

### VP1 gene sequencing and phylogenetic analysis

A High Fidelity one-step RT-PCR Kit (TaKaRa, Japan) was used to amplify the target gene segment. According to the provided protocol, the total volume per PCR was 50 µl which included 10 pmol of each of the primers F: 5’-GCRTGCAATGAYTTCTCWGT-3’, R: 5’-GCICCIGAYTGITGICCRAA-3’. The reverse transcription reaction was performed by incubation at 50°C for 30 min and terminated by incubation at 94 °C for 2 min, then followed by 30 cycles at 94 °C for 30 s, 45 °C for 30 s , 72°C for 1 min. The products were detected in 1.5% Ethidium Bromide pre-stained agarose gel after electrophoresis. The products were purified and then used for sequencing. VP1 gene DNA sequences of the EV isolates were compared to the National Center for Biotechnology Information (NCBI) database through BLAST. Based on the sequences of the VP1gene, phylogenetic analysis was done by using the Mega 5.10 software.

## Results

### Detection and typing of the EVs

A total of 1180 samples were used for EV detection through cell culture during the study period of 2002-2012 (Figure S1A-S1D). From 826 CSF samples and 354 stool samples, 13.9% (115/826) and 31.1% (110/354) were positive for EV. During these 11years, the positive EV isolation ratio was fluctuated between 4.8% in 2007 and 35.2% in 2003 (Table 1).

**Table 1:** Enterovirus isolation and serotyping result during the period of 2002-2012.

Year	Positive cases No. (%,)	Enterovirus serotypes												
		A9	B1	B2	B3	B4	B5	E3	E4	E6	E9	E14	E25	E30
2002	13 (17.6%)				1		2							10
2003	19 (35.2%)													19
2004	49 (34.3%)													49
2005	5 (12.2%)				1		1		2				1	
2006	5 (5.7%)		1		1		2	1						
2007	8 (4.8%)				2		6							
2008	32 (16.8%)		2		14		4		1	5				6
2009	35 (28.0%)		3		2		22							8
2010	25 (21.0%)			1		7	2			8		1		6
2011	7 (19.4%)													7
2012	27 (18.6%)	2			1		1	1		2	3		1	16
Total	225 (19.1%)	2	6	1	22	7	40	2	3	15	3	1	2	121

A: Coxsackievirus A; B: Coxsackievirus B; E: Echovirus.

The EV isolates were further used for serotyping through neutralization assay. The serotype of these isolates distributed in thirteen types, including 2 (Coxsackievirus A9), 6 (Coxsackievirus B1), 1 (Coxsackievirus B2), 22 (Coxsackievirus B3), 7 (Coxsackievirus B4), 40 (Coxsackievirus B5), 2 (Echovirus 3), 3 (Echovirus 4), 15 (Echovirus 6), 3 (Echovirus 9), 1 (Echovirus 14), 2 (Echovirus 25), 121 (Echovirus 30). As shown in the Table 1, the dominate EV serotypes prevailed in different years were significantly different. We can find that from 2002-2004, 2011-2012 the Echovirus 30 was the dominate serotype, while it was not detected from 2005-2007. 

With the development of the molecular detection method for the EV, we used the real-time RT-PCR for EV identification in 2012 (Figure S1E). The result showed 24.1% (35/145) positive detection ratio, while the virus isolation result showed 18.6% (27/145) positive ratio for the detection of EV. Except for the same virus detected by these two methods, extra 3 Echovirus 9, 1 Echovirus 16 and 4 Echovirus 30 associated cases were identified by the molecular detection method.

The clinical features of the EAE cases were analyzed according to the data collected from 2008-2012. For totally 393 EAE cases, including 260 males and 133 females, 88.5% (348/393) showed the symptom of fever, 59.5% (234/393) for upper respiratory symptoms, 89.8% (353/393) for headache, 76.1% (299/393) for nausea, 86.8% (341/393) for vomiting, 5.6% (22/393) altered consciousness. Convulsion, history of high grad fever and neck stiffness, which were the most common clinical features of JE were significantly rarely found in EAE.

### VP1 gene based phylogenetic analysis

The VP1 gene of the EV was amplified by the conventional RT-PCR (Figure S1F). The amplification products were purified, sequenced and then used for phylogenetic analysis. A total of 135 EV strains, which covered all the isolated serotypes were used for sequencing analysis. The whole length of the VP1 genes from all these isolates was from 834 to 918. After comparison with VP1 genes from different reference EV strains respectively, the homologous was 76.7%-85.0% (nucleotide) and 91.1%-98.1% (amino acid), which met the serotype identification criteria for homologous serotypes (Table S1). The molecular typing results showed all EV isolates were belonged to the human EVB specie. Base on the VP1 gene, the homologous comparison results for the isolates from the same serotypes were 79.6%-98.0% (nucleotide), 95.2%-99.8% (amino acid) (Table S1). The Echovirus 6 isolates showed the biggest divergence, including 20.4% (nucleotide), 4.8% (amino acid). And the Coxsackievirus B3 isolates showed the smallest divergence, including 2.0% (nucleotide), 0.4% (amino acid). The serotype Echovirus 30 isolates, which dominated for the EVAE of Zhejiang showed the highest nucleotide identity (90.2%-99.0%) with the Echovirus 30 isolates from Jiangsu (FDJS/18/03) and Shandong (SD/06530/06).

**Figure 1:**
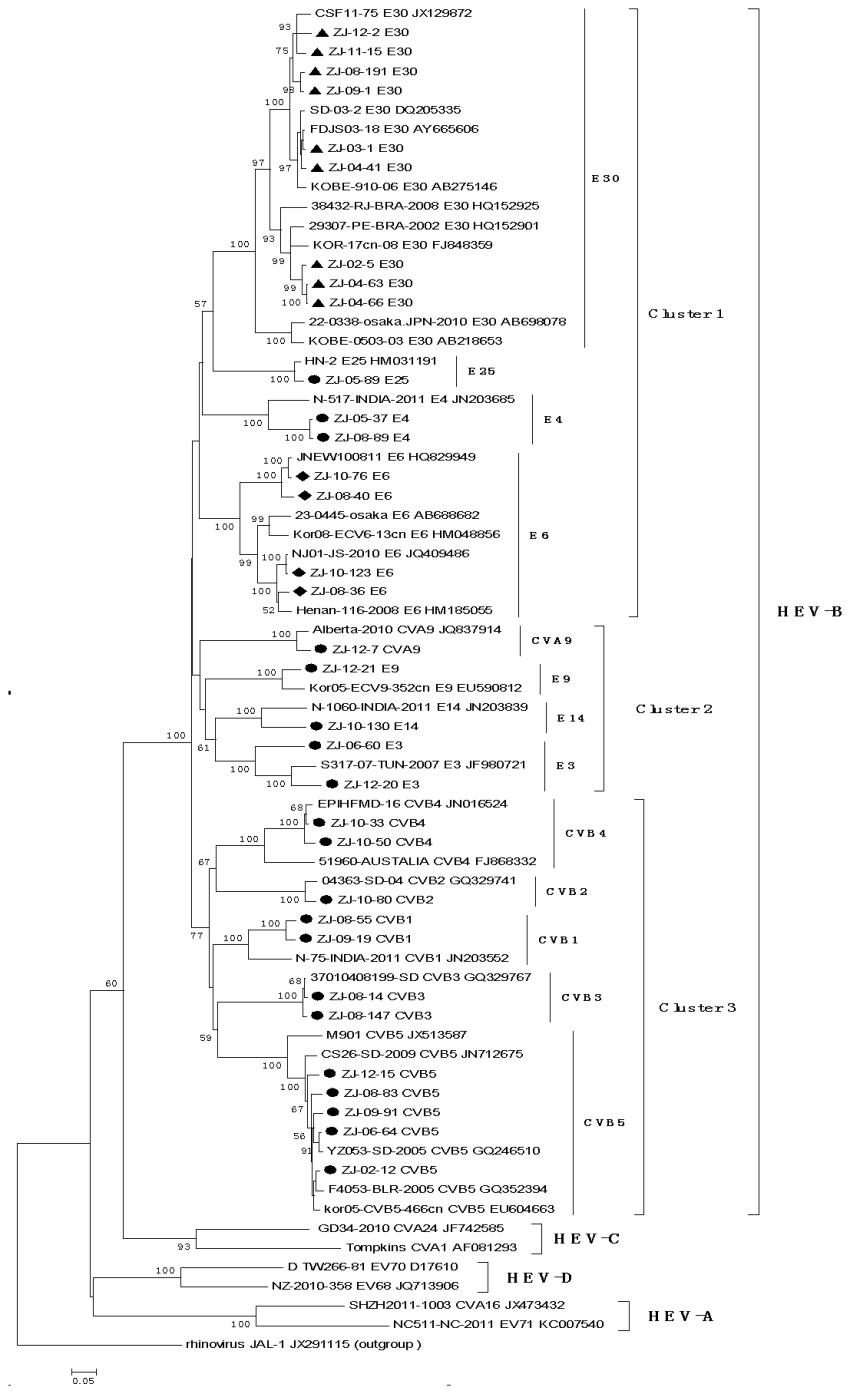
Phylogenetic analysis of the enterovirus isolates based on the whole VP1 gene sequences. The tree was constructed by using the neighbor-joining method. Significance of phylogenies was investigated by bootstrap analysis with 1,000 pseudoreplicate data sets. Bootstrap values of are indicated on the tree. ●, ▲and ◆: Enterovirus isolates from this study. All reference sequences are named by using the sample name/serotype/GenBank accession numbers. Rhnovirus JAL-1 was used as out group control. The scale bar indicates nucleotide substitutions per site. The bracket on the right indicates the enterovirus species.

Based on the VP1 gene sequences, phylogenetic analysis for the EVs of this study was done by comparison with all available VP1 gene sequences from the Genbank. From the constructed phylogenetic tree (Figure 1), we can find all the Human EV diverged into four groups (Human EV A-D). All the isolates from Zhejiang belonged to the human EVB and distributed in three clusters, including E30, E25, E4, E6 isolates in cluster I, E3, E9, E14 and CVA9 isolates in cluster II, and the other isolates in cluster III. Though the Echovirus 30 isolates from different years distributed in the same cluster, they were further diverged into two significant genotypes. The E30 isolates were most closely related to the stains from Jiangsu (FDJS03-18), Shandong (SD-03-2), Japan (KOBE-910-06), Korea (KOR-17cn-08) (Figure 1).

## Discussion

According to the previous study, EV viruses are regarded as significant important etiological agents for the encephalitis, which is an inflammation of the brain parenchyma associated with substantial morbidity and mortality [7,8]. However, the epidemiologic and clinical features of the EAE in east China have not been characterized before. According to our long-period (2002-2012) and large-number cases (1165) study, we confirmed that the EV was also an important etiology for the encephalitis cases here. With the detection of 1180 samples, 13.9% CSF and 31.1% stool samples were positive for EV. Results also showed that the positive EV isolation ratio fluctuated greatly (4.8% to 35.2%) in different years. It was suggested that the EV was not always the main pathogen for the unknown etiological encephalitis. Echovirus 30 was found to be the periodically predominate serotype for the EAE in the periods of 2002-2004 and 2010-2012, which indicated that specific serotypes of enterovirus can also only be predominating in a particular period, others would introduced and cause epidemics. As we can also find that no Echovirus 30 was detected from 2005-2007, and Coxsackievirus B3, B5 was the predominating serotype for 2008 and 2009. And in 2010, Coxsackievirus B4 was found to cause epidemic for EAE for the first time here (Table 1).

Based on the phylogenetic analysis of the EV isolates through VP1 gene comparison, it was found that all the isolates from Zhejiang belonged to the human EVB. The sequences of the VP1 genes were more closely related among the isolates from the near areas. The dominate serotype Echovirus 30 were found diverged into two significant genotypes. What interesting is that the two genotypes happened to present in the same year. After analysis the data further, we also found that the positive ratios for these two genotypes were significantly different. It suggested that the virulence of these two genotypes of Echovirus 30 can be also quite different. Sequences of these viruses have been compared. The change of the antigen structure and the difference of the virulence would need to be determined further.

During the surveillance, we applied the real-time RT-PCR for the detection of EV from CSF and stool samples in 2012, and compared with the classic virus isolation method. The positive detection ratio (24.1%) of real time RT-PCR was higher than the virus isolation (18.6%). After the molecular typing through the sequence of the VP1 gene, we found the detection result of real time RT-PCR showed good accordance with result of the virus isolation. Except for the same virus detected by this two methods, extra Echovirus 9, 16, 30 were identified by the molecular detection method. It means that the new molecular detection method is more sensitive, and it was also thought to be more stable and easy manipulation.

According to the clinic features of the EAE observed from this study, ratios for altered sensorium and mortality were significantly less than JE. Fever was the most common clinical feature of encephalitis, but the EAE did not show the high grade fever as JE. These clinic features can be significantly useful for the clinic distinguish of the EAE. More importantly, clinical surveillance for the EAE cases would provide crucial information for EAE prevention and evaluation of the potential possibility for EAE out break.

It is the first report of the long-period investigation of unknown etiological encephalitis cases in Zhejiang, China. The data of this study would contribute greatly to the control of EAE, and predicting of the potential EV serotype prevailing for encephalitis in the future. 

## Supporting Information

Figure S1 **Enteroviruses isolation, qRT-PCR identification and VP1 gene amplification.** A: Normal Hep-2 cell control. B: Cytopathic effects of the enterovirus growing in Hep-2 cells. C: Normal RD cell control. D: Cytopathic effects of the enterovirus growing in RD cells. E: Enterovirus RNA detection through real time qRT-PCR. 1: Positive amplification control; 2-3: Positive amplification results with the detection of the enterovirus RNA from CFS and stool samples; 4: Negative control. F: Conventional PCR amplification result of the VP1 gene. M: Marker; 1-2: PCR amplification products of the target VP1 genes from enterovirus isolates; 3-4: Negative control.
(TIF)

Table S1 **Sequence analysis result of the enterovirus isolates from 2002-2012.** A: Coxsackievirus A; B: Coxsackievirus B; E: Echovirus.
(DOC)
