# Supplementary figures and images for: Novel and Predominant Pathogen Responsible for the Enterovirus-Associated Encephalitis in Eastern China
Source: PLoS One. 2013 Dec 30;8(12):e85023. doi: 10.1371/journal.pone.0085023 (PMC3875553; doi:10.1371/journal.pone.0085023)

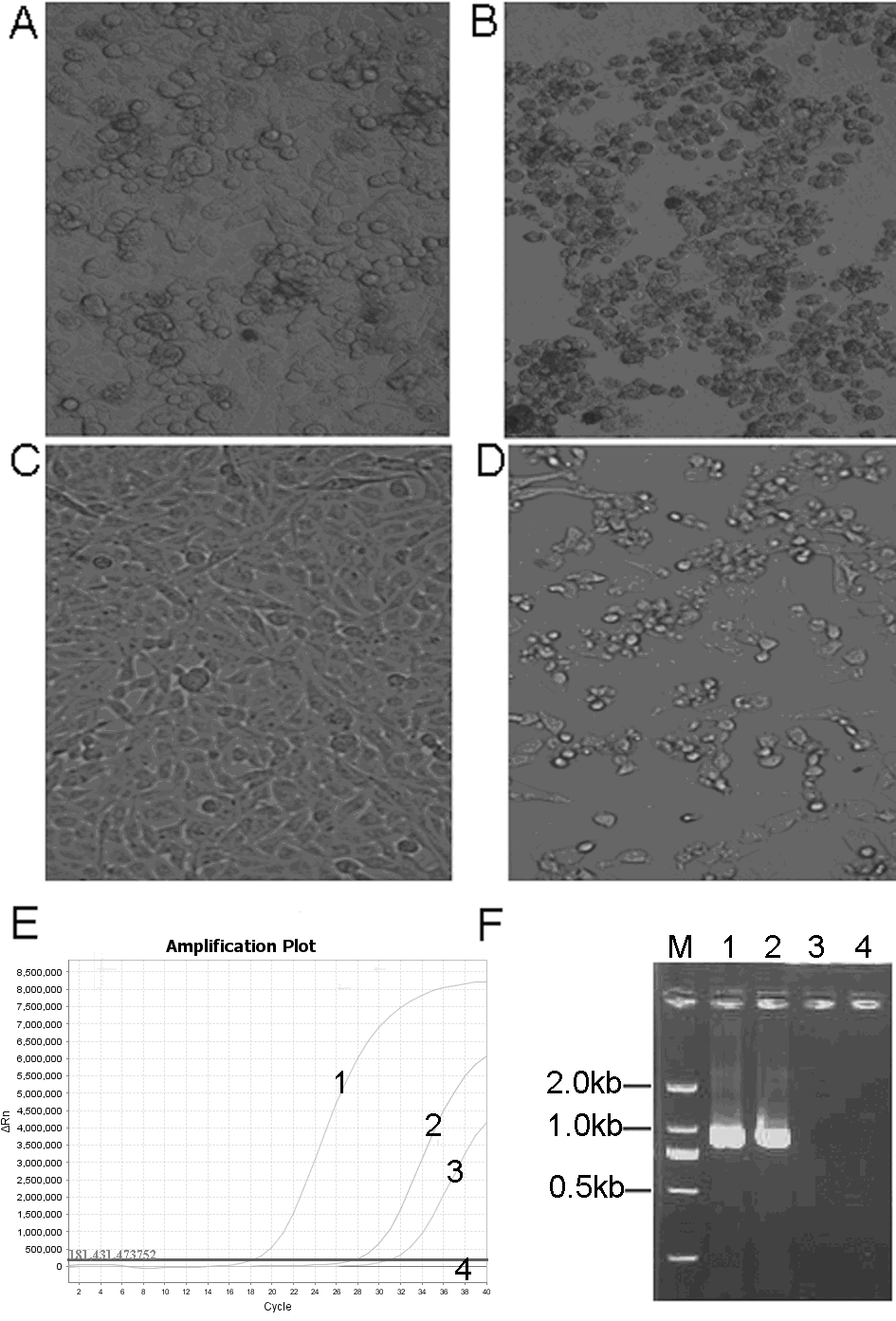

Supplement: Figure S1 — Enteroviruses isolation, qRT-PCR identification and VP1 gene amplification. A: Normal Hep-2 cell control. B: Cytopathic effects of the enterovirus growing in Hep-2 cells. C: Normal RD cell control. D: Cytopathic effects of the enterovirus growing in RD cells. E: Enterovirus RNA detection through real time qRT-PCR. 1: Positive amplification control; 2-3: Positive amplification results with the detection of the enterovirus RNA from CFS and stool samples; 4: Negative control. F: Conventional PCR amplification result of the VP1 gene. M: Marker; 1-2: PCR amplification products of the target VP1 genes from enterovirus isolates; 3-4: Negative control. (TIF) [file pone.0085023.s001.tif]
