# Supplementary material for: Novel and Predominant Pathogen Responsible for the Enterovirus-Associated Encephalitis in Eastern China
Source: PLoS One. 2013 Dec 30;8(12):e85023. doi: 10.1371/journal.pone.0085023 (PMC3875553; doi:10.1371/journal.pone.0085023)
Supplement: Table S1 — Sequence analysis result of the enterovirus isolates from 2002-2012. A: Coxsackievirus A; B: Coxsackievirus B; E: Echovirus. (DOC) [file pone.0085023.s002.doc]

| Serotype | No. of isolates | Reference strains | Length of VP1gene (bp) | Comparison between isolates | | Comparison with reference strains | |
| --- | --- | --- | --- | --- | --- | --- | --- |
|  |  |  |  | nt (%) | aa (%) | nt (%) | aa (%) |
| A9 | 2 | Griggss | 906 | 95.3 | 98.0 | 80.7~81.1 | 91.1~92.4 |
| B1 | 5 | Conn-5 | 834 | 96.5~99.4 | 99.8~100.0 | 78.7~78.9 | 92.4 |
| B2 | 1 | Ohio-1 | 846 | NA | NA | 81.7 | 98.1 |
| B3 | 4 | Nancy | 852 | 98.0~98.2 | 99.6~100.0 | 79.0~79.1 | 96.5 |
| B4 | 5 | JVB | 852 | 96.5~99.2 | 99.3~100.0 | 85.0~85.4 | 97.9~98.6 |
| B5 | 25 | Faulkner | 849 | 93.9~100.0 | 99.3~99.7 | 80.9~81.9 | 96.5~97.2 |
| E3 | 2 | Morrisey | 876 | 80.0 | 97.5 | 80.6~82.5 | 97.2~97.5 |
| E4 | 3 | Pesacek | 843 | 94.3~98.2 | 99.6 | 81.4~81.6 | 95.4~95.7 |
| E6 | 15 | Charles | 867 | 79.6~94.3 | 95.2~99.7 | 76.7~77.9 | 92.5~96.6 |
| E9 | 3 | Hill | 918 | 95.8~96.1 | 97.6~99.3 | 82.8~84.3 | 94.6~95.9 |
| E14 | 1 | Tow | 894 | NA | NA | 79.4 | 96.7 |
| E25 | 2 | JV-4 | 876 | 93.2 | 97.3 | 79.3~80.6 | 93.2 |
| E30 | 67 | Bastianni | 876 | 85.5~100.0 | 96.2~100.0 | 81.6~84.7 | 92.5~93.5 |
